# Short‐term probiotic supplementation affects the diversity, genetics, growth, and interactions of the native gut microbiome

**DOI:** 10.1002/imt2.253

**Published:** 2024-12-16

**Authors:** Xin Shen, Hao Jin, Feiyan Zhao, Lai‐Yu Kwok, Zhixin Zhao, Zhihong Sun

**Affiliations:** ^1^ Key Laboratory of Dairy Biotechnology and Engineering, Ministry of Education Inner Mongolia Agricultural University Hohhot China; ^2^ Key Laboratory of Dairy Products Processing, Ministry of Agriculture and Rural Affairs Inner Mongolia Agricultural University Hohhot China; ^3^ Inner Mongolia Key Laboratory of Dairy Biotechnology and Engineering Inner Mongolia Agricultural University Hohhot China; ^4^ Collaborative Innovative Center for Lactic Acid Bacteria and Fermented Dairy Products, Ministry of Education Inner Mongolia Agricultural University Hohhot China

## Abstract

The precise mechanisms through which probiotics interact with and reshape the native gut microbiota, especially at the species and genetic levels, remain underexplored. This study employed a high‐dose probiotic regimen of *Bifidobacterium animalis* subsp. *lactis* [200 billion colony forming units (CFU)/day] over 7 days among healthy participants. Weekly fecal samples were collected for metagenomic sequencing analysis. We found that probiotic intake can significantly enhance the diversity of the gut microbiome and impact single nucleotide variations, growth rates, and network interactions of the resident intestinal bacteria. These adaptive changes in the gut microbiota indicate the swift evolutionary responses of native bacteria to the ecological disturbance presented by probiotic supplementation. Notably, the microbial community appears to undergo rapid and multifaceted ecological adjustments, potentially preceding longer‐term evolutionary changes. This knowledge lays the groundwork for further exploration into the mechanisms underlying probiotic‐mediated modulation of the gut microbiome, highlighting the necessity of encompassing ecological and evolutionary perspectives in the design and optimization of probiotic applications.

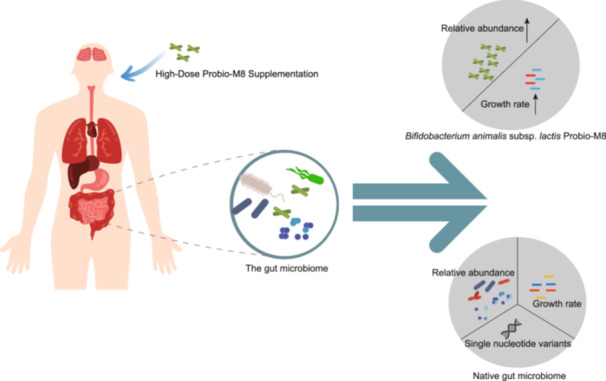

The human gut harbors a highly diverse and dynamic microbiome that plays a crucial role in maintaining overall health and physiological homeostasis [1]. Probiotics, defined as live microorganisms that provide health benefits when administered in adequate amounts, represent a promising strategy for supporting gut health. Both preclinical and clinical studies have demonstrated the potential of probiotic supplementation in managing various health conditions. However, the effects of probiotics can vary significantly among individuals, highlighting the importance of tailoring probiotic interventions to individual microbiome characteristics.

Exogenous probiotic strains, upon entering the gut, can influence the native microbiota not only at the species level but also at the strain level [2, 3, 4]. Since the functional contributions of the gut microbiome are largely determined by strain‐level activity, it is essential to analyze microbial growth dynamics at this finer resolution to fully understand how probiotics regulate the gut microbiota. Currently, the most commonly used dosage of probiotics in clinical studies ranges from 10^6^ to 10^9^ colony forming units (CFU) per day [5, 6, 7], with the use of higher doses becoming increasingly common. However, there remains a significant knowledge gap regarding the persistence and colonization ability of these probiotics and whether these traits are reliably translated into enhanced health benefits, particularly in healthy individuals. Importantly, it is still unclear how high‐dose probiotic interventions affect the host gut microbiome at finer taxonomic and genetic levels, as well as the nature of their interactions within the gut ecosystem.

To address these gaps, we selected a well‐characterized probiotic strain, Probio‐M8, which has been demonstrated to have positive effects on gut health [8, 9], and administered it at a high dose of 200 billion CFU per day in a short‐term intervention study involving healthy individuals. We performed deep metagenomic sequencing on multiple fecal samples to investigate the impact of the probiotic on gut microbiota from both macro and micro diversity perspectives (Figure 1A and Table S1). Additionally, we constructed an ecological regulation network centered on Probio‐M8. Our findings suggest that the microbial community undergoes rapid and multifaceted ecological adjustments, which may precede longer‐term evolutionary changes.

**FIGURE 1:**
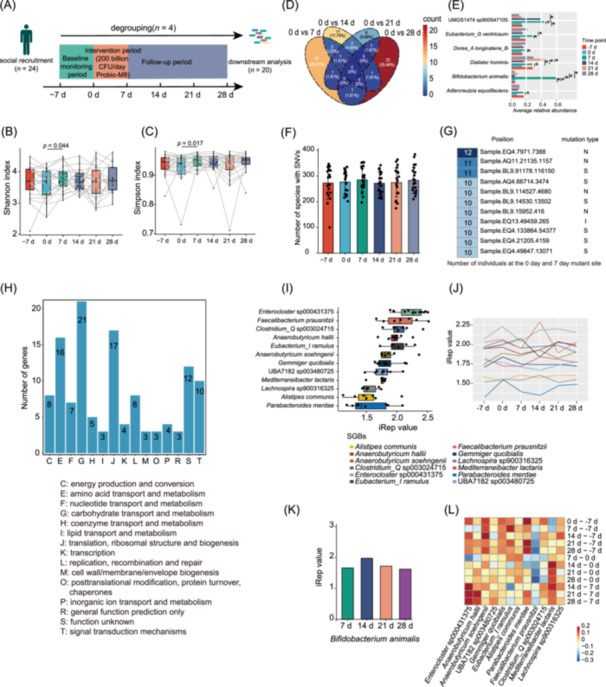
Intervention trial design and changes in key microbial properties of the gut microbiota after probiotic intervention. (A) Schematic overview of the intervention trial timeline. (B) Boxplots depicting the distributions of Shannon diversity indices for the participants' gut microbiomes at baseline (−7 d), before and after the probiotic intervention (0 d, 7 d), and during the follow‐up period (14 d, 21 d, and 28 d). (C) Boxplots depicting the distributions of Simpson diversity indices for the participants' gut microbiomes at baseline (−7 d), before and after the probiotic intervention (0 d, 7 d), and during the follow‐up period (14 d, 21 d, and 28 d). The gray lines connect the data points for the same individual across different time points. (D) Venn plot depicting the distribution of differentially abundant species‐level genome bins (SGBs) between baseline (0 d) and various time points after the probiotic intervention (7 d, 14 d, 21 d, and 28 d). (E) Horizontal bar chart showing the mean relative abundance of the differential SGBs across different time points during the intervention trial. Statistical differences between time points were assessed using the Wilcoxon rank‐sum test, with *p* < 0.05 considered significant. (F) Bar chart illustrating the number of bacterial species presenting single nucleotide variants (SNVs) during the intervention trial. Error bars represent standard deviation (SD). (G) The heatmap shows gene locations and corresponding mutation types at shared sites. (H) Bar chart shows the number of genes and the corresponding functional types of unshared sites. (I) Boxplots illustrating the index of replication (iRep) values for bacterial species to which growth rates were annotated in at least one‐third of the samples collected before intervention (0 d). The iRep metric serves as a proxy for the growth rate of individual microbial species within the gut ecosystem. (J) Line plots show changes in iRep values at different time points for bacterial species whose growth rates were annotated in at least one‐third of the samples collected before intervention (0 d). (K) The mean iRep value of *Bifidobacterium animalis* at different time points after the probiotic Probio‐M8 ingestion. (L) Difference in iRep values between two time points for bacteria annotated to the irep index in at least one‐third of samples collected before intervention (0 d). The color scale represents the magnitude of the difference, with warmer (reddish) colors indicating increases, while cooler (bluish) colors signifying decreases between the two time points.

## SHORT‐TERM HIGH‐DOSE PROBIO‐M8 ADMINISTRATION ENHANCES GUT MICROBIOTA DIVERSITY BUT INDUCES LIMITED MICROBIOTA COMPOSITION CHANGES

To accurately evaluate the abundance and impact of probiotics within the gut microbiota, it is crucial to generate comprehensive and high‐resolution data. To achieve this, we employed a sequencing depth that was 5–10 times greater than that of typical studies [10]. Following rigorous quality control and the removal of host DNA, we obtained a total of 4.98 TB (terabyte) of high‐quality sequencing data, averaging 41.53 ± 5.20 GB (gigabyte) per sample, which ensured the robustness and reliability of downstream analyses. Leveraging this extensive metagenomic data set, we assembled 4878 high‐quality genome bins (completeness > 80%, contamination < 5%), and ultimately identified 507 species‐level genome bins (SGBs) with an average nucleotide identity of 95% and a genome alignment rate of 30%.

We then assessed the overall impact of the probiotic intervention on the macro‐diversity of the gut microbiota by analyzing Shannon and Simpson diversity indices. Initially, no significant differences were observed between the two groups on the 7 days before probiotic intervention and on day 0 (−7 d and 0 d), indicating stable gut microbiota diversity before intervention. Following the administration of Probio‐M8, both diversity indices showed a significant increase (*p* < 0.05; Figure 1B,C, and Table S2), demonstrating that the intervention effectively enhanced the gut microbiota diversity. However, this increase in alpha diversity was transient as it remained stable thereafter, suggesting that while short‐term and high‐dose probiotic intake can enhance diversity, it may not be sufficient to induce persistent changes within the complex gut ecosystem.

To further investigate the impact on gut microbiota composition, we conducted principal coordinates analysis (based on the Bray‐Curtis dissimilarity) and ANOSIM (analysis of similarities). These analyses indicated no significant changes in the overall gut microbiota composition following intervention (Figure S1A,B). Thus, while short‐term and high‐dose administration of Probio‐M8 effectively enhanced microbiota diversity, it did not significantly alter the community composition of the resident microbiota.

To identify gut microbiota species with altered abundance during the intervention period, we conducted a taxonomic differential abundance analysis, which identified several significantly affected species, including *Dialister hominis*, *Bifidobacterium animalis*, and *Eubacterium_G ventriosum* (Figure 1D,E, and Table S3). Among them, *Adlercreutzia equolifaciens*, UMGS1474 sp900547105, and *Eubacterium_G ventriosum* increased in abundance after the intervention, which decreased after stopping probiotic intake, while *Dorea_A longicatena_B* and *Dialister hominis* also increased in abundance after the intervention, but their abundance fluctuated afterward. Notably, *Bifidobacterium animalis* demonstrated a significant increase in abundance at day 7 compared to days −7 and 0 (Figure 1E). Analysis using average nucleotide identity (ANI) confirmed that the increased abundance was primarily due to the administered Probio‐M8 strain. However, this effect was transient, as the abundance of *Bifidobacterium animalis* was returned to baseline levels following the cessation of probiotic intervention. These findings indicate that Probio‐M8 cannot maintain a high abundance in the gut of most individuals without continuous supplementation, highlighting the need for sustained probiotic intake to achieve prolonged effects.

## SHORT‐TERM HIGH‐DOSE PROBIO‐M8 ADMINISTRATION IMPACTS THE MICRODIVERSITY AND GROWTH DYNAMICS OF THE GUT MICROBIOTA

To further understand the subtle genetic shifts within the gut microbiota that may be driven by external factors, it is crucial to consider the role of evolutionary pressures. These pressures can induce microdiversity within microbial communities, influencing the overall structure and function of the gut ecosystem [11]. We subsequently examined single nucleotide variants (SNVs) within the gut microbiota. Our findings revealed a nonsignificant increase in the number of species harboring SNVs following Probio‐M8 administration at day 7 compared to the baseline period (−7 d and 0 d; Figure 1F). We further analyzed species with a read mapping coverage of 0.4 at least, comparing base changes before and after the intervention (0 d and 7 d). SNV loci were classified as shared or non‐shared, based on whether more than 50% of the loci differed between pre‐ and post‐intervention. In total, 11 shared loci and 2442 non‐shared loci were identified. Of these, six loci were synonymous mutations. Functional annotation based on the database of Clusters of Orthologous Genes (COGs) revealed functions, including myosin‐crossreactive antigen, alpha‐l‐fucosidase, and argininosuccinate synthase (Figure 1G, and Table S4). The 2442 non‐shared loci were distributed across 120 genes, 21 of which were associated with carbohydrate transport and metabolism (Figure 1H).

The in situ bacterial replication rate was assessed using the iRep metric, which estimates genome replication rates from metagenomic datasets, thereby providing insights into bacterial growth and the proportion of live bacteria in the gut microbiota [12]. We used the iRep algorithm to evaluate the impact of Probio‐M8 intervention on the growth rate profile of bacterial species within the gut. An iRep value closer to 2 signifies a higher replication rate. Our analysis revealed that different gut microbes responded variably to Probio‐M8 intake (Figure 1I,J). For example, *Enterocloster* sp000431375 exhibited the greatest iRep value, but its growth rate decreased after the probiotic intervention. However, it is important to note that despite the high iRep value, it remained a non‐dominant species within the gut microbiota. This observation underscores the fact that swift bacterial replication within complex microbial communities may not necessarily lead to a rise in population size due to various factors impacting the absolute bacterial abundance, such as the original species abundance and other ecological interactions like competition, predation, and cellular mortality. Furthermore, the replication rate of bacteria at different time points was studied, and it was found that the replication rate of different strains was different after probiotic consumption (Figure 1J). Unfortunately, our study on growth rate was contingent solely on the assessment of genome replication dynamics, leaving the cellular mortality rate undisclosed. Interestingly, upon analyzing *Bifidobacterium animalis* specifically, we observed an increased replication rate following Probio‐M8 consumption, with the iRep value almost reaching 2 at 14 d (Figure 1K). To provide a comprehensive visualization of the iRep metric analysis, we employed a heatmap, which revealed varied changes in growth rates across different gut microbes (Figure 1L).

Overall, these results demonstrate that the short‐term and high‐dose intake of the Probio‐M8 probiotic formulation not only modulated the relative abundance of specific gut bacterial taxa but also influenced the microdiversity and growth dynamics of the indigenous gut microbiota.

## PROBIO‐M8 MODIFIES GUT MICROBIAL ECOLOGY WITHOUT GENETIC CHANGES IN DOMINANT SPECIES

**FIGURE 2:**
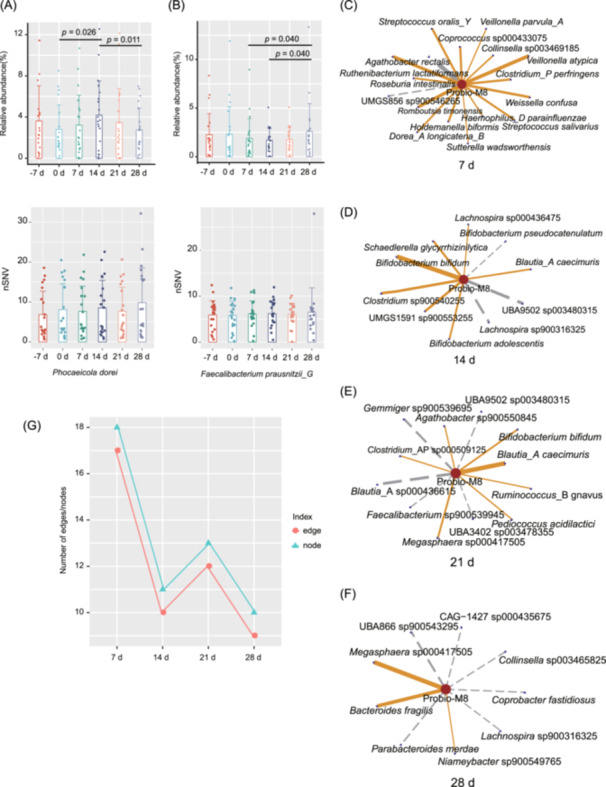
Effects of probiotic intervention on the relative abundance, genetic diversity, and network interactions of gut microbiota. (A) This figure presents the changes in the relative abundance and the normalized number of single nucleotide variations (nSNVs; lower panel) for the *Phncaeicola dorei* in the participants' gut microbiome. (B) This figure presents the changes in the relative abundance and the normalized number of single nucleotide variations (nSNVs; lower panel) for the *Faecalibacterium prausnitzii_G* in the participants' gut microbiome. The data of various time points are shown: before the probiotic intervention (−7 d and 0 d), after the intervention (7 d), and during the follow‐up period (14 d, 21 d, and 28 d). The Wilcoxon rank‐sum test was used to evaluate the statistical significance of the differences between time points, with *p* < 0.05 considered significant. (C–F) Network interaction plots illustrate the correlations between Probio‐M8 and other gut resident bacteria at various time points (7 d, 14 d, 21 d, and 28 d, respectively) after the ingestion of the probiotic Probio‐M8. The orange lines indicate positive correlations, while the gray lines represent negative correlations. The thickness of the lines corresponds to the strength of the correlation, with thicker lines denoting stronger associations. The size of the circle represents the number of nodes; the larger the circle, the more nodes it indicates. (G) The number of nodes and edges of the correlation network between Probio‐M8 and other gut resident bacteria at various time points (7 d, 14 d, 21 d, and 28 d, respectively) after the ingestion of the probiotic.

To assess whether the Probio‐M8 intervention led to genetic changes within the gut microbial community, we focused our analysis on the SNVs of the top eight dominant species. These species were selected based on their prevalence and significant influence within the microbiome, making them prime candidates for detecting genetic changes in response to ecological shifts. Considering that SNV frequency is positively correlated with sequencing depth, we normalized the number of SNVs (nSNVs) to sequencing depth using the formula: nSNVs = number of SNVs/sequencing depth per sample. Despite significant post‐interventional shifts in the relative abundance of certain dominant species, such as *Faecalibaterium prausnitzii*_G, *Phocaeicola dorei*, *Escherichia coli*, *Alistipes putredinis*, and *Faecalibaterium prausnitzii*, compared to baseline (Figure 2A,B, Figure S1C, *p* < 0.05), no significant differences were observed in the nSNV across these species at any time point. These findings indicate that while the Probio‐M8 intervention led to notable ecological shifts within the gut microbial community, these changes were not accompanied by significant genetic adaptations within the dominant species over the study period. Additionally, our results align with a prior meta‐analysis, which reported limited ecological changes after probiotic intake, while noting extensive genetic alterations within individual gut microbes [11]. These comparisons highlight the complexity of microbial responses to probiotics, indicating that while ecological shifts may occur rapidly, significant genetic adaptations may require more prolonged exposure or specific environmental conditions. Further research is needed to explore these dynamics more comprehensively, especially across different populations and extended time scales.

## PROBIO‐M8 AS A KEY MODULATOR OF GUT MICROBIAL INTERACTION NETWORKS

To assess the impact of Probio‐M8 intervention on the interaction dynamics within the gut microbial community, we employed Spearman correlation analyses to evaluate the changes in inter‐bacterial relationships over time, focusing on shifts in interaction strength and patterns. Using a correlation coefficient threshold of 0.5 (*p* < 0.05), we visualized the microbial interactions by constructing a correlation matrix heatmap. Our analysis revealed that Probio‐M8 administration influenced the strength and complexity of interactions within the gut microbial network (Figure S1D).

A more detailed examination of correlations involving Probio‐M8 provided additional insights. Visualization of the correlation‐based interaction network demonstrated that Probio‐M8 exhibited the highest number of associations with other resident gut microbes at day 7, indicating an expansion of its interaction network following the intervention (Figure 2C). Quantification of the nodes and edges in the network centered around Probio‐M8 showed that both metrics peaked at day 7 but declined subsequently during follow‐up, suggesting that the enhanced connectivity was transient (Figure 2D–G, and Table S5). Our findings indicate that Probio‐M8 administration transiently enhanced interconnectivity within the gut microbial community, underscoring the dynamic ecological responses of the gut microbiota to probiotic interventions. The observed modulation of microbial interaction networks provides valuable insights into the transient nature of probiotic‐induced shifts in microbial ecology.

In summary, this study employed deep metagenomic sequencing to evaluate in situ changes in the gut microbiota in response to high‐dose probiotic intervention. Our findings provide critical insights into the intricate interactions between probiotics and the indigenous gut microbial community. We observed that short‐term administration of Probio‐M8 enhanced gut microbial diversity and influenced key microbial properties, including nucleotide variation, growth dynamics, and inter‐bacterial interactions, highlighting the significant impact of probiotics on the ecological balance of the gut ecosystem. Notably, our results indicate that gut microbes rapidly adapt to probiotic‐induced pressures, with ecological changes often preceding genetic adaptations, underscoring the complexity and multifaceted nature of the microbiome response. Although the study is limited by its sample size, these findings offer valuable perspectives on the transient nature of probiotic‐induced shifts in the gut microbiota. Further large‐scale and multi‐population studies are warranted to deepen our understanding of probiotic‐microbiome interactions and to support the development of targeted and precision probiotics for optimizing gut health.

## AUTHOR CONTRIBUTIONS

**Xin Shen**: Visualization; writing—original draft. **Hao Jin**: Writing—original draft; validation. **Feiyan Zhao**: Conceptualization; methodology; software. **Lai‐Yu Kwok**: Writing—review and editing. **Zhixin Zhao**: Validation; data curation. **Zhihong Sun**: Project administration; formal analysis; supervision.

## CONFLICT OF INTEREST STATEMENT

The authors declare no conflict of interest.

## ETHICS STATEMENT

This study protocol was reviewed and approved by the Ethical Committee of the Affiliated Hospital of Inner Mongolia Medical University [Approval NO.KY (2020013)], and the clinical trial has been registered with the Chinese Clinical Trials Registry (ChiCTR Identifier: ChiCTR2000039167).

## Supporting information


**Figure S1:** Changes in gut microbiota structure, abundance, genetic diversity, and association after probiotic intervention.

**Table S1:** Participants' information.
**Table S2:** Differences in the alpha diversity of gut microbiota across time points.
**Table S3:** Significantly differential species‐level genome bins (SGBs) identified between time points.
**Table S4:** Shared loci and COG (database of Cluster of Orthologous Genes) annotation results.
**Table S5:** Correlation between *Bifidobacterium animalis* subsp. *lactis* Probio‐M8 and SGBs.

## Data Availability

The data that supports the findings of this study are available in the supplementary material of this article. The sequencing data generated in this study was made available in the CNGB Sequence Archive (CNSA) (https://db.cngb.org/cnsa/) of China National Genebank DataBase (CNGBdb) with accession number CNP0005636. The data and scripts used are saved in GitHub https://github.com/shenx08/M8-high-dose.git. Supplementary materials (methods, figures, tables, graphical abstract, slides, videos, Chinese translated version, and update materials) may be found in the online DOI or iMeta Science http://www.imeta.science/.
