# Supplementary material for: Short‐term probiotic supplementation affects the diversity, genetics, growth, and interactions of the native gut microbiome
Source: Imeta. 2024 Dec 16;3(6):e253. doi: 10.1002/imt2.253 (PMC11683461; doi:10.1002/imt2.253)
Supplement: Supplementary file 1 — Figure S1: Changes in gut microbiota structure, abundance, genetic diversity, and association after probiotic intervention. [file IMT2-3-e253-s001.docx]

**Supporting information to**

**Short-term probiotic supplementation affects the diversity, genetics, growth, and interactions of the native gut microbiome**

**Running Title:** Probiotic supplementation affects native gut microbiome

Xin Shen^1,2,4#^, Hao Jin^1,2,4#^, Feiyan Zhao^1,2,3^, Lai-Yu Kwok^1,2,3^, Zhixin Zhao^1,2,3^, Zhihong Sun^1,2,4*^

^1^Key Laboratory of Dairy Biotechnology and Engineering, Ministry of Education, Inner Mongolia Agricultural University, Hohhot 010018, China.

^2^Key Laboratory of Dairy Products Processing, Ministry of Agriculture and Rural Affairs, Inner Mongolia Agricultural University, Hohhot 010018, China.

^3^Collaborative Innovative Center for Lactic Acid Bacteria and Fermented Dairy Products, Ministry of Education, Inner Mongolia Agricultural University, Hohhot 010018, China.

^4^Inner Mongolia Key Laboratory of Dairy Biotechnology and Engineering, Inner Mongolia Agricultural University, Hohhot 010018, China.

^#^ These authors contributed equally: Xin Shen, Hao Jin

*Correspondence: [sunzhihong78@163.com](mailto:sunzhihong78@163.com) (Zhihong Sun)

**Methods**

## Ethics approval and consent to participate

The study was conducted by the principles of the Declaration of Helsinki. This study protocol was reviewed and approved by the Ethical Committee of the Affiliated Hospital of Inner Mongolia Medical University [Approval NO.KY (2020013)]. Before participation, all participants were provided with detailed information about the study objectives, procedures, and potential risks and benefits. Written informed consent was obtained from each participant. This clinical trial has been registered with the Chinese Clinical Trials Registry (ChiCTR Identifier: ChiCTR2000039167)

## Trial design and subject recruitment

This was a 5-week longitudinal study designed to investigate the effects of short-term, high-dose probiotic supplementation on the gut microbiome of healthy adults.

Subjects were recruited through the whole society. Inclusion criteria were: (1) age between 25 to 85 years, with a balanced male-to-female ratio; (2) no history of major illness; (3) no long-term antibiotic use; and (4) willingness to provide written informed consent. Exclusion criteria were: (1) failure to meet the inclusion criteria; (2) poor adherence to the study protocol; (3) withdrawal from the study; and (4) use of oral probiotics products other than the study supplement.

Based on these criteria, a total of 24 volunteers were recruited to participate in the study. After a 7-day baseline period (-7 to 0 days), participants received a daily dose of 200 billion CFU of the probiotic supplement Probio-M8 (Jinhua Yinhe Biotechnology Co., Ltd.) for 7 days (intervention period, days 0-7). Fecal samples were collected from all participants at weekly intervals from baseline (-7 d, 0 d) to the end of the follow-up periods (7 d, 14 d, 21 d, and 28 d; Figure 1A). Twenty participants completed the entire 5-week study protocol (Table S1).

During the study, all participants maintained their normal dietary habits and abstained from consuming alcoholic beverages. Fecal samples collected from the participants were stored on dry ice during transportation to the laboratory and then frozen at -80 °C for subsequent analysis.

## Metagenomic DNA extraction and sequencing

Fecal DNA was extracted from all collected samples using the QIAamp Fast DNA Stool Mini kit (QIAGEN GmbH, Hilden, Germany), strictly following the manufacturer's protocol. The extracted DNA samples were then purified using AMpure magnetic beads (Beckman Coulter, Inc., Brea, CA, USA) to remove potential inhibitors. The concentration and purity of the resultant DNA samples were assessed using the Qubit® dsDNA Assay Kit (Thermo Fisher Scientific, Waltham, MA, USA) and Nanodrop spectrophotometer, respectively. DNA integrity was further evaluated by 1% agarose gel electrophoresis. Only DNA samples meeting the quality control criteria, with an *OD*_260/280_ ratio between 1.8 and 2.0 and a concentration of ≥ 20 ng/μL, were considered acceptable for downstream sequencing. In total, 120 qualified DNA samples (from six different time points) underwent paired-end sequencing on the Illumina NovaSeq 6000 platform (Tianjin NovoGold Science and Technology Co., Ltd., Tianjin, China) to generate metagenomic data.

## Quality control of metagenome data

Following the removal of host-derived DNA sequences, the raw metagenomic reads were assembled into contigs using the MEGAHIT software [1]. The assembled contigs were then subjected to binning using the variational autoencoders for metagenomic binning (VAMB) software to obtain draft metagenome-assembled genomes (MAGs) [2]. The reads from each sample were mapped back to the assembled contigs using the BWA-MEM aligner. The alignment data were processed using the SamTools (v.1.9) software and the jgi_summarize_bam_contig_depths function in MetaBAT2 to calculate the read depth, and use CheckM assess the completeness and contamination levels of the generated MAGs. Only high-quality MAGs with completeness greater than 80% and contamination levels below 5% were retained for further analysis. Finally, the high-quality MAGs were clustered using the dRep software to identify the most representative genomes, compiling a list of 507 species-level genome bins (SGBs), which was subsequently used for downstream taxonomic and functional analyses (Figure 1B).

## Taxonomic annotation and relative abundance of SGBs

To taxonomically annotate the 507 SGBs recovered from the metagenomic data, we employed the Kraken2 classifier and the National Center for Biotechnology Information non-redundant nucleotide sequence databases. For functional annotation, we used the Prodigal software to predict genes within the SGB sequences and then performed sequence similarity searches against the UniProt Knowledgebase (UniProtKB, release 2020.11) using the blastp algorithm with default parameters. The relative abundance of each SGB was calculated using the CoverM tool (https://github.com/wwood/CoverM), which estimated the coverage of each SGB based on the mapped metagenomic reads. These relative abundance values were then used for further bioinformatics and statistical analysis.

## Species-level diversity and growth rate analysis

To assess the species-level diversity of the bacterial community within our metagenomic dataset, we employed InStrain [3], which allowed us to detect SNVs within each of the identified high-quality SGBs. Additionally, we utilized the Index of Replication (iRep) metric to estimate the genome replication and growth rates for high-quality SGBs. This approach provides insights into the metabolic activity and growth dynamics of the different bacterial populations represented in the metagenome.

## Statistical analysis and data visualization

All statistical analyses were carried out using the R software (v.4.2.3). The bar chart is presented as mean ± standard deviation. To calculate the relative abundance of the identified SGBs, we utilized the vegan and optparse packages, the Wilcoxon rank-sum test was used to calculate the differences between groups. To assess the beta-diversity patterns across samples collected at different time points, we calculated Bray-Curtis distance matrices using the vedist package in R. We then performed Anosim analyses (with 999 permutations) to evaluate the statistical significance of sample differences. Additionally, we conducted Spearman rank correlation analyses to investigate the relationships between the relative abundances of the different bacterial taxa. Heatmap, Venn diagram and Correlation Network Diagram were generated using the OmicStudio tools at <https://www.omicstudio.cn/tool>, and other graphical presentations were created using R software and Adobe Illustrator

**Reference**

1. Li, Dinghua, Chi-Man Liu, Ruibang Luo, Kunihiko Sadakane, Tak-Wah Lam. 2015. “MEGAHIT: an ultra-fast single-node solution for large and complex metagenomics assembly via succinct de Bruijn graph.” *Bioinformatics* 31: 1674-1676. <https://doi.org/10.1093/bioinformatics/btv033>

2. Nissen, [Jakob Nybo](https://pubmed.ncbi.nlm.nih.gov/?term=Nissen+JN&cauthor_id=33398153), Joachim Johansen, Rosa Lundbye Allesøe, Casper Kaae Sønderby, Jose Juan Almagro Armenteros, Christopher Heje Grønbech, Lars Juhl Jensen, et al. 2021. “Improved metagenome binning and assembly using deep variational autoencoders.” *Nat Biotechnol* 39: 555-560. <https://doi.org/10.1038/s41587-020-00777-4>

3. Olm, Matthew R, Alexander Crits-Christoph, Keith Bouma-Gregson, Brian A Firek, Michael J Morowitz, Jillian F Banfield. 2021. “inStrain profiles population microdiversity from metagenomic data and sensitively detects shared microbial strains.” *Nat Biotechnol* 39: 727-736. <https://doi.org/10.1038/s41587-020-00797-0>


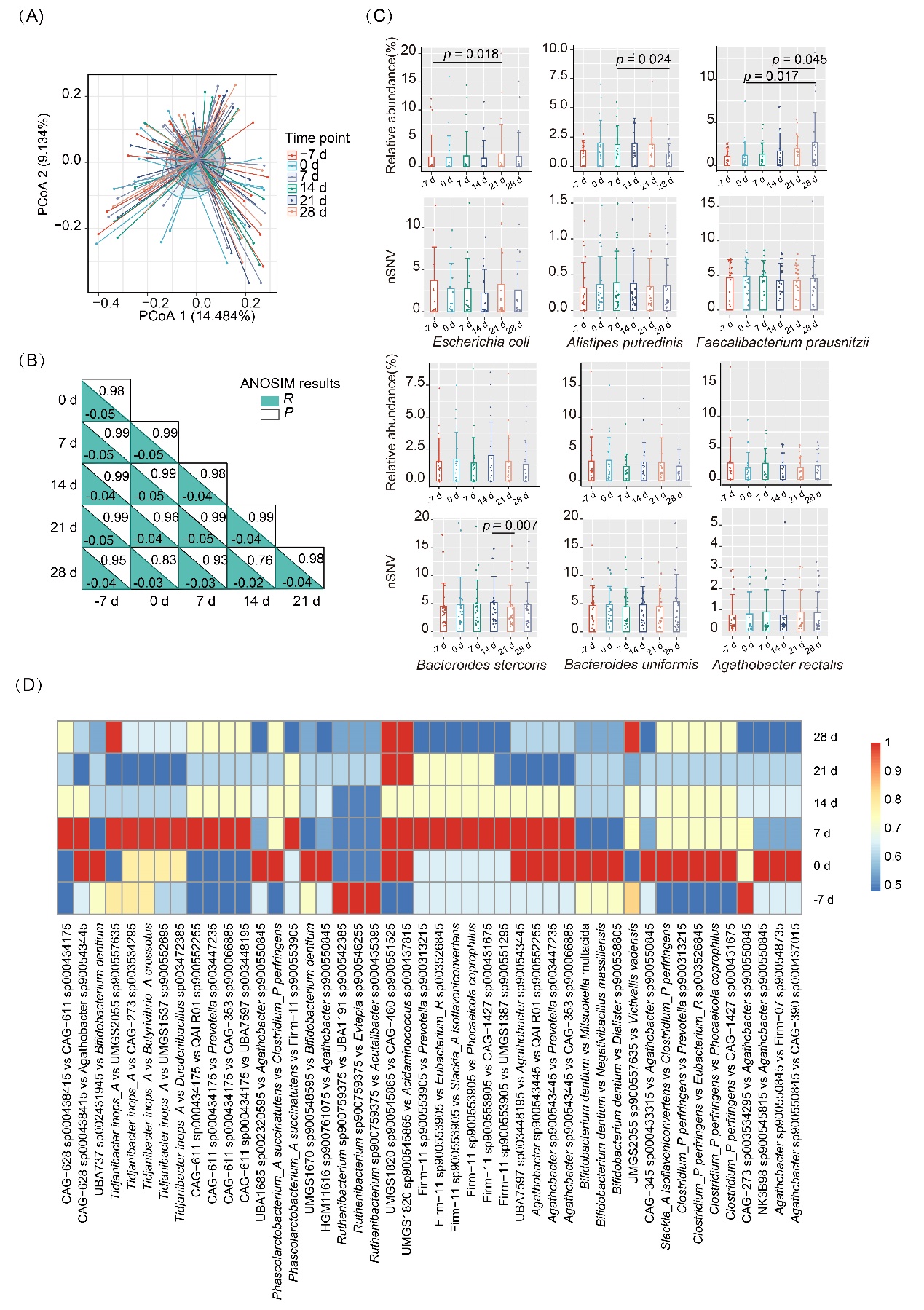


**Figure S1 Changes in gut microbiota structure, abundance, genetic diversity, and association after probiotic intervention.** (A) Principal coordinate analysis (PCoA) score plot based on Bray-Curtis distance, which illustrates the overall compositional shifts in the gut microbiome throughout the intervention. (B) The results of the analysis of similarities (ANOSIM) test are presented, where the *R* and *P* values (shown in green and white triangles in the dissimilarity matrix, respectively) indicate the statistical significance of the pairwise differences between the gut microbiome compositions at the various time points. (C) Relative abundance and genetic diversity of dominant gut microbes. This figure presents the changes in the relative abundance and the normalized number of single nucleotide variations (nSNVs; lower panel) for the most abundant species-level genome bins (SGBs) in the participants' gut microbiome. The data of various time points are shown: before the probiotic intervention (-7 d and 0 d), after the intervention (7 d), and during the follow-up period (14 d, 21 d, and 28 d). The Wilcoxon rank-sum test was used to evaluate statistical significance of the differences between time points, with *p* < 0.05 considered significant. (D) Spearman’s correlation heatmap depicting the relationships between specific species-level genome bins (SGBs) identified at different time points. The color scale represents Spearman’s *rho* correlation coefficient, ranging from blue (strong) to red (very strong) positive correlation. This analysis reveals the dynamic nature of microbial co-occurrence patterns in response to the probiotic intervention.
